# Case Report: A *de novo* Variant of *CRYGC* Gene Associated With Congenital Cataract and Microphthalmia

**DOI:** 10.3389/fgene.2022.866246

**Published:** 2022-05-27

**Authors:** Yu Peng, Yu Zheng, Zifeng Deng, Shuju Zhang, Yilan Tan, Zhengmao Hu, Lijuan Tao, Yulin Luo

**Affiliations:** ^1^ Department of Ophthalmology & Pediatrics Research Institute of Hunan Province, Hunan Children’s Hospital, Changsha, China; ^2^ Pediatrics Research Institute of Hunan Province, Hunan Children’s Hospital, Changsha, China; ^3^ Department of Ophthalmology, Hunan Children’s Hospital, Changsha, China; ^4^ Center for Medical Genetics & Hunan Key Laboratory of Medical Genetics, School of Life Sciences, Central South University, Changsha, China

**Keywords:** congenital cataract, crystallin, *CRYGC*, microphthalmia, whole-exome sequencing

## Abstract

**Background:** Congenital cataract is one of the most common causes of blindness in children. A rapid and accurate genetic diagnosis benefit the patients in the pediatric department. The current study aims to identify the genetic defects in a congenital cataract patient without a family history.

**Case presentation:** A congenital cataract patient with microphthalmia and nystagmus was recruited for this study. Trio-based whole-exome sequencing revealed a *de novo* variant (c.394delG, p.V132Sfs*15) in *CRYGC* gene. According to the American College of Medical Genetics and Genomics (ACMG) criteria, the variant could be annontated as pathogenic.

**Conclusion:** Our findings provide new knowledge of the variant spectrum of *CRYGC* gene and are essential for understanding the heterogeneity of cataracts in the Chinese population.

## Background

Congenital cataract is visible at birth or during the first decade of life; it is usually diagnosed by red light reflex, ophthalmoscopy examination and ocular color doppler ultrasound. Congenital cataract is one of the most common causes of blindness in children, with an estimated prevalence of 1–6 cases per 10,000 live births (Santana and Waiswo, 2011). About 8.3%–25% of congenital cataract cases present Mendelian inheritance; autosomal dominant inheritance pattern is the most common, but autosomal recessive and X-linked patterns have also been reported (Merin and Crawford, 1971; Francois, 1982; Zhong et al., 2017).

Inherited cataracts are genetically heterogeneous. With the development of WGS techniques, more and more cataract-related genes have been mapped and identified. So far, there are at least 49 loci and 37 genes have been identified for inherited isolated forms of cataracts according to OMIM (https://www.ncbi.nlm.nih.gov/omim/). These genes can be roughly grouped into four categories: crystallins, membrane proteins, cytoskeletal proteins, and DNA/RNA-banding proteins (Shiels and Hejtmanick, 2015). Crystallins are a kind of water-soluble protein that compose about 90% of lenticular protein mass and maintain the transparency of the lens (Hoehenwarter et al., 2006). They are divided into three major classes, α-, β-, and γ-crystallins. The α-crystallins belong to the small heat shock protein (HSP20) family, accounting for up to 50% of the total soluble protein of the lens (Bhat, 2003). Furthermore, they act as chaperones by binding partially unfolded lens βγ-crystallins to prevent their aggregation and thus maintain the transparency of the lens (Bhat, 2003). The βγ-crystallins are a superfamily of proteins with a “Greek key” motif unit base. Generally, the βγ-crystallins are supposed to be the essential structural proteins of the lens, but their exact function is still not fully understood (Jaenicke and Slingsby, 2001; Bhat, 2003; Slingsby and Wistow, 2014). Human γ-crystallins include six Cryg genes (*CRYGA*, *CRYGB*, *CRYGC*, *CRYGD*, *CRYGN*, and *CRYGS*); among them, variants of *CRYGC*, *CRYGD*, *CRYGS*, and *CRYGB* have been reported to be associated with congenital cataract (Heon et al., 1999; Stephan et al., 1999; Sun et al., 2005; AlFadhli et al., 2012).

In this study, a novel 1-bp deletion (c.394delG) in *CRYGC* gene was detected in a congenital cataract patient by trio-based whole-exome sequencing.

### Case Presentation

**FIGURE 1:**
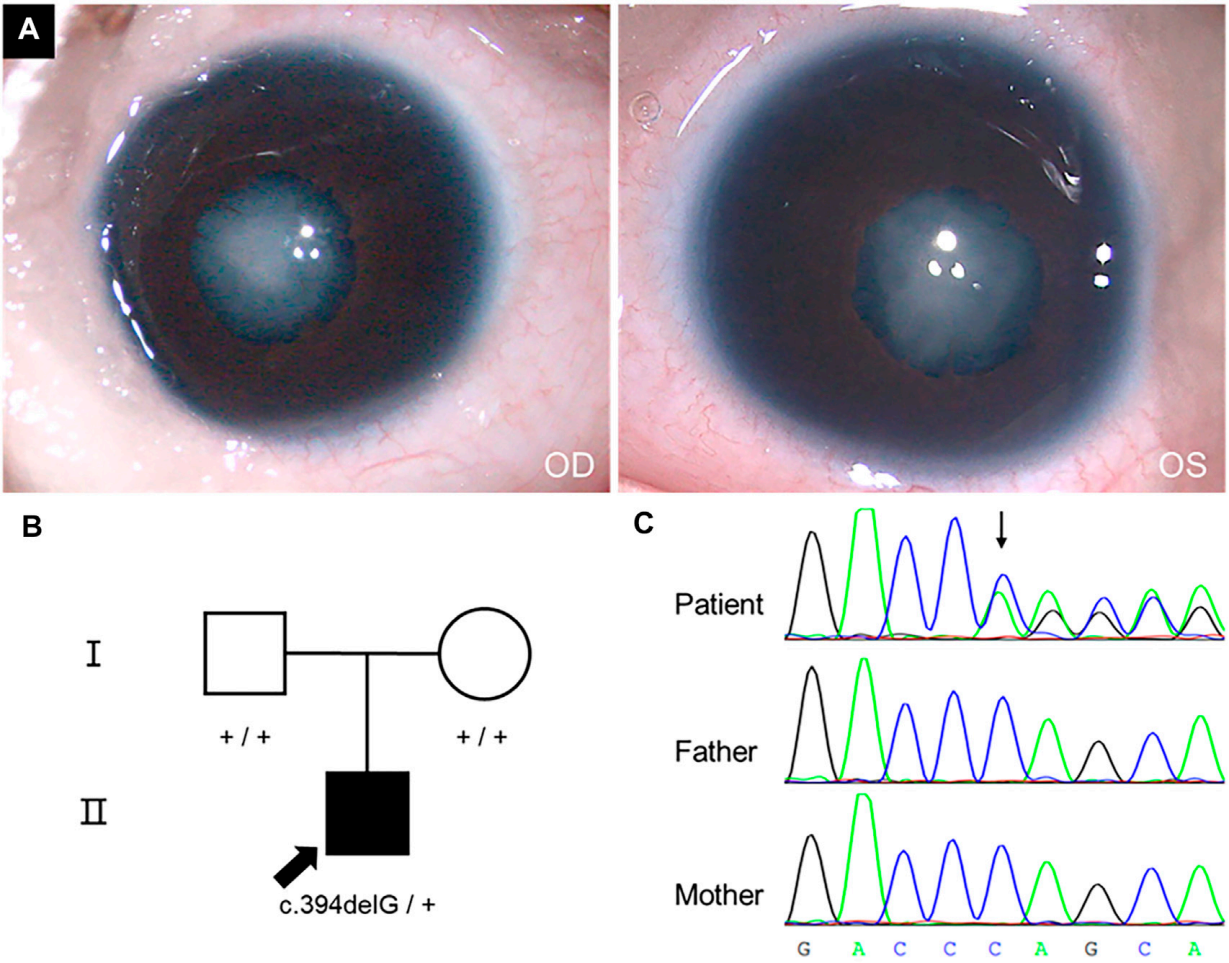
Phenotype, pedigree, and Sanger sequencing results. **(A)** Cataract phenotype and pupils with irregular borders of the proband were shown. **(B)** The pedigree of a congenital cataract trios family. **(C)** c.394delG variant in *CRYGC* gene.

The patient was examined at three months old because the pupil area of both eyes was found to be white for 15 days. He had poor light tracing reactions and no family history of cataracts. An ophthalmological exam revealed bilateral phacoscotasmus (C5), shallow anterior chamber, persistent pupillary membrane, invisible fundus, and nystagmus (Figure 1A). His corneas were transparent and had a diameter of 7.5 mm. The axial lengths of his eyes were 15.13 mm (OD) and 15.05 mm (OS), respectively. The intraocular pressures (IOP) were 10.2 mmHg (OD) and 14.0 mmHg (OS). Ultrasonography showed no alterations other than the opacified lens and reduced axial lengths.

A diagnosis of total cataracts and bilateral microphthalmia was made. Vitrectomy and lensectomy *via* anterior approach, posterior capsulorhexis, and peripheral iridectomy were performed on his both eyes. On postoperative one day, the IOP of the patient was 11 and 13 mmHg in the right and left eyes, respectively. Levofloxacin eye drops, tobramycin and dexamethasone eye drops, and tropicamide phenylephrine eye drops were used four times per day. 1 month after surgery, refractive correction in diopters (dpt) was +22.00 dpt −1.00 × 180 for the right eye and +22.00 dpt −1.00 × 180 for the left eye with spectacles. At the same time, the patient began amblyopia training under the guidance of doctors and parents.

## Methods

### Genomic DNA Preparation

DNA was isolated from peripheral blood using DNA Isolation Kit (Blood DNA Kit V2, CW2553). Concentrations were determined on a Qubit fluorometer (Invitrogen, Q33216) using Qubit dsDNA HS Assay Kit (Invitrogen, Q32851). Agarose gel (1%) electrophoresis was performed for quality control.

### Whole-Exome Sequencing

1 μg of the isolated DNA was sheared into about 200 bp sized fragments using Bioruptor UCD-200 (Diagenode). 3 μl of the sheared DNA was electrophoresed in a 2% agarose gel to confirm the presence of fragments of the desired size range. DNA libraries were prepared with KAPA Library Preparation Kit (Kapa Biosystems, KR0453) following the manufacturer’s instructions. The libraries were estimated with Qubit dsDNA HS Assay kit (Invitrogen, Q32851). The hybridization of pooled libraries to the capture probes and remove non-hybridized library molecules were carried out by Agilent SureSelectXT2 Target Enrichment System. DNA libraries were sequenced on the Illumina Novaseq. 6000 platform (Illumina, San Diego, CA, United States) as paired-end 150-bp reads. Sample dilution, flowcell loading and sequencing were performed according to the Illumina specifications. Each sample yielded more than 10 Gb of raw data; over 95% of bases had a Phred quality score >20. The mean coverage was ×100 of the genome and the minimum coverage of ×10 was about 99%.

### Data Analysis

FastQC (http://www.bioinformatics.babraham.ac.uk/projects/fastqc/) tool was used to evaluate reads quality, and our in-house script was used to filter low-quality reads. The sequenced raw reads in FastQ file format were preprocessed using Trim Galore (version 0.6.4, http://www.bioinformatics.babraham.ac.uk/projects/trim_galore/) to remove adapter-contaminated ends and low-quality bases with Phred scores < 20. Reads with > 5N bases, > 40% low-quality bases, or trimmed lengths < 30 bp were also removed. Subsequently, the quality passed reads were subsequently mapped to the human reference sequence (version: hg19) by alignment tool Burrows Wheeler Aligner (BWA, v0.7.17) (Li and Durbin, 2009). SNPs and small InDels were generated with Genome Analysis Toolkit (GATK, v3.8) (McKenna et al., 2010). The parent-child relationship was identified by King software (v2.2.7) (Manichaikul et al., 2010) to confirm the *de novo* variant.

### Sanger Sequencing

Sanger sequencing was used to validate the variant through the filtering procedures. Primers were designed by the Primer3 program (http://frodo.wi.mit.edu/).

## Result

WES yielded 14.7, 10.3, and 13.2 Gb data from genomes of proband, father, and mother, respectively. Totally, 17,823 nonsynonymous SNVs and 549 Indels were identified. Considering the patient has no family history, we checked *de novo* variants and recessive inherit variants at first. We identified 93 recessive inherit variants (including homozygous and compound heterozygous variants, Max MAF < 0.05), involving 54 genes. But none of these genes was associated with cataracts. In addition, there were 24 *de novo* variants (Max MAF < 0.005) involving 19 genes in the proband. A *de novo* frameshift variant c.394delG (hg19: chr2:208993058) was identified in *CRYGC* gene (NM_020989) through our filter pipeline. The variant would cause a frameshift from the 132nd codon and prematurely terminate at the 147th codon if a mutant protein was produced (p.V132Sfs*15). Sanger sequencing confirmed that the variant is heterozygous in the proband but absent from his parents (Figure 1C). The relationships between the three samples were confirmed (Supplementary Table S1). Furthermore, the variant was absent in the gnomAD exomes or genomes (http://gnomad.broadinstitute.org). Therefore, the c.394delG variant could be categorised as pathogenic according to the American College of Medical Genetics and Genomics (ACMG) criteria (Richards et al., 2015) (PVS1+PS2+PM2).

## Discussion

To date, a total of 32 variants in *CRYGC* gene have been reported to be associated with congenital cataract (Heon et al., 1999; Ren et al., 2000; Santhiya et al., 2002; Gonzalez-Huerta et al., 2007; Devi et al., 2008; Yao et al., 2008; Zhang et al., 2009; Kumar et al., 2011; Guo et al., 2012; Li et al., 2012; Kondo et al., 2013; Reis et al., 2013; Gillespie et al., 2014; Prokudin et al., 2014; Li et al., 2016; Ma et al., 2016; Patel et al., 2017; Sun et al., 2017; Zhong et al., 2017; Astiazaran et al., 2018; Li et al., 2018; Zhang et al., 2019; Zhuang et al., 2019; Berry et al., 2020; Taylan Sekeroglu et al., 2020; Fernandez-Alcalde et al., 2021; Karahan et al., 2021; Rechsteiner et al., 2021), but there were few reports about the *de novo* mutations. In 2017, Zhong et al. reported a frameshift mutation (p.Asp65ThrfsX38) which might be *de novo* (Zhong et al., 2017). In 2021, Rechsteiner et al. reported a *de novo* mutation p.Glu107GlyfsX56, which causes cataracts and microphthalmia (Rechsteiner et al., 2021), and Fernández-Alcalde et al. reported a *de novo* mutation p.Leu145GlyfsX5 (Fernandez-Alcalde et al., 2021). In the present study, a *de novo* frameshift variant (c.394delG, p.V132Sfs*15) was identified in *CRYGC* gene as the cause of a patient with congenital cataract and microphthalmia.

CRYGC has a two-domain beta-structure, folded into four similar Greek key motifs (GKM); like all γ-crystallins, it has the highest intrachain symmetry (Blundell et al., 1981). The high degree of symmetry may contribute to the stability of γ-crystallins (Blundell et al., 1981). *CRYGC* variants in GKMs may disrupt the symmetrical structure, which changes the intra- or inter-molecular interactions, possibly leading to destabilisation and aggregation, respectively (Zhong et al., 2017). The variant p.V132Sfs*15 occurred at the beginning of GKM4 (129-171aa), leading to a frameshift and premature termination, disrupting the entire GKM4.

According to Cat-Map (Shiels et al., 2010) (https://cat-map.wustl.edu/, last updated on October 2021), the most common phenotype caused by *CRYGC* variants was nuclear cataracts, followed by lamellar and pulverulent cataracts. The missense variants p.F6S and p.R168W had been reported to be associated with either nuclear or lamellar cataracts (Santhiya et al., 2002; Gonzalez-Huerta et al., 2007; Devi et al., 2008; Astiazaran et al., 2018). It seems that there was no particular connection between cataract phenotypes and variant sites. Inherited cataracts could be isolated or associated with other ocular signs, including microcornea/microphthalmia, eye movement disorders (nystagmus, strabismus, amblyopia), or refractive errors. There 15 variants were reported to cause cataracts and additional ocular signs among all the 32 reported *CRYGC* variants. Microcornea was the most common additional ocular sign (Zhang et al., 2009; Guo et al., 2012; Reis et al., 2013; Patel et al., 2017; Sun et al., 2017; Zhong et al., 2017; Rechsteiner et al., 2021). The phenotypic heterogeneity could be due to unidentified modifier genes (Astiazaran et al., 2018) or some unknown mechanisms in which CRYGC takes part during eye development. For example, proteomics research showed that the CRYGC and some other crystallins are highly expressed in the human cornea (Subbannayya et al., 2020), indicating that these genes might involve in the cornea morphogenesis and transparency.

Next-generation DNA sequencing technologies could identify the precise genetic cause in about 45%–75% of congenital cataract families. For example, testing of WES in 11 cataract families by Kandaswamy et al. determined a genetic cause in 6 families (55%) (Kandaswamy et al., 2020). A recent study on inherited eye diseases found that WGS (through 100,000 Genomes Project) had a diagnostic yield of 44.7% (17/38) for congenital cataract families (Jackson et al., 2020). In the past few years, it has been reported that testing of a targeted gene panel (115 genes) in 36 bilateral cataracts patients identified a genetic cause in 75% of cases (Gillespie et al., 2014). However, another research using the same panel established a genetic diagnosis in 50% of congenital cataract cases (Lenassi et al., 2020). A rapid and accurate genetic diagnosis in the pediatric department helps patients understand their cause of disease, make clinical decisions, carry on the instruction for procreation, or even look for therapeutic schemes. In the current study, a genetic cause was identified in a three-month-old congenital cataract patient. He underwent cataract surgery immediately after diagnosis and had a good prognosis.

## Conclusion

In conclusion, we have identified a novel frameshift variant, c.394delG, p.V132Sfs*15, within the *CRYGC* gene in a congenital cataract boy. Our findings provide new knowledge of the variant spectrum of *CRYGC* and are essential for understanding the heterogeneity of cataracts in the Chinese population.

## Data Availability

The datasets presented in this study can be found in online repositories. The names of the repository/repositories and accession number(s) can be found below: (BankIt2557536 BSeq#1 OM912449).
