# Supplementary material for: Case Report: A de novo Variant of CRYGC Gene Associated With Congenital Cataract and Microphthalmia
Source: Front Genet. 2022 May 27;13:866246. doi: 10.3389/fgene.2022.866246 (PMC9198712; doi:10.3389/fgene.2022.866246)
Supplement: Supplementary file 1 [file Table1.docx]

**Table S1 Relationship confirm by King software**

| ID1 | ID2 | Kingship* | Relationship |
| --- | --- | --- | --- |
| Proband | Father | 0.1907 | 1st-degree |
| Proband | Mother | 0.1911 | 1st-degree |
| Mother | Father | 0.0103 | Unrelated |

*An estimated kinship coefficient range >0.354, (0.177, 0.354), (0.0884, 0.177) and (0.0442, 0.0884) corresponds to duplicate/MZ twin, 1st-degree, 2nd-degree, and 3rd-degree relationships respectively (https://www.kingrelatedness.com/).
